# Increasing thermal stability and improving biodistribution of VEGFR2-binding affibody molecules by a combination of in silico and directed evolution approaches

**DOI:** 10.1038/s41598-020-74560-5

**Published:** 2020-10-23

**Authors:** Rezan Güler, Siri Flemming Svedmark, Ayman Abouzayed, Anna Orlova, John Löfblom

**Affiliations:** 1 Department of Protein Science, School of Engineering Sciences in Chemistry, Biotechnology and Health, KTH Royal Institute of Technology, Stockholm, Sweden; 2 Department of Medicinal Chemistry, Uppsala University, Uppsala, Sweden; 3 Science for Life Laboratory, Uppsala University, Uppsala, Sweden; 4 Research Centrum for Oncotheranostics, Research School of Chemistry and Applied Biomedical Sciences, Tomsk Polytechnic University, Tomsk, Russia

**Keywords:** Proteins, Biotechnology, Molecular medicine

## Abstract

The family of vascular endothelial growth factor (VEGF) ligands and their interactions with VEGF receptors (VEGFRs) play important roles in both pathological and physiological angiogenesis. Hence, agonistic and antagonistic ligands targeting this signaling pathway have potential for both studies on fundamental biology and for development of therapies and diagnostics. Here, we engineer VEGFR2-binding affibody molecules for increased thermostability, refolding and improved biodistribution. We designed libraries based on the original monomeric binders with the intention of reducing hydrophobicity, while retaining high affinity for VEGFR2. Libraries were displayed on bacteria and binders were isolated by fluorescence-activated cell sorting (FACS). In parallel, we used an automated sequence- and structure-based in silico algorithm to identify potentially stabilizing mutations. Monomeric variants isolated from the screening and the in silico approach, respectively, were characterized by circular dichroism spectroscopy and biosensor assays. The most promising mutations were combined into new monomeric constructs which were finally fused into a dimeric construct, resulting in a 15 °C increase in melting temperature, complete refolding capability after heat-induced denaturation, retained low picomolar affinity and improved biodistribution profile in an in vivo mouse model. These VEGFR2-binding affibody molecules show promise as candidates for further in vivo studies to assess their suitability as molecular imaging and therapeutic agents.

## Introduction

Vascular endothelial growth factor receptor 2 (VEGFR2) is a transmembrane receptor tyrosine kinase that is important in both physiological and pathological angiogenesis. Its activation promotes vascular permeability, as well as cell migration and proliferation^1,2^. The different VEGF family ligands bind to VEGFRs and act as agonists by inducing dimerization of the receptors which in turn leads to subsequent phosphorylation of the intracellular kinase domains and signaling. The role of angiogenesis in diseases such as age-related macular degeneration, diabetic retinopathy, and tumor progression, has made it a prevalent target for therapeutic strategies^3^. Various therapeutics have been developed for anti-angiogenic treatment by targeting VEGFR2 signaling. Bevacizumab, an anti-VEGF monoclonal antibody (mAb) that binds and neutralizes human VEGF isoforms is approved for glioblastoma, colorectal cancer and several other cancer types^4,5^. Aflibercept, a recombinant Fc-linked decoy receptor that is based on fusion of subdomains from VEGFR1 and VEGFR2, binds VEGF isoforms as well as the placenta growth factor (PIGF)^6,7^ and has been approved for treatment of metastatic colorectal cancer in combination with chemotherapy, and for wet macular degeneration. Ramucirumab, a mAb that blocks the ligand-binding site on VEGFR2 has been approved as a monotherapy for gastroesophageal and metastatic gastric cancer and several other cancer forms^8^. Here we report on the engineering and evaluation of an affibody-based VEGFR2 binder that has a similar mechanism of action as ramucirumab.

Affibody molecules are small (58 residues) three-helical affinity proteins, originally derived from one of the immunoglobulin-binding domains of staphylococcal protein A^9,10^, which are engineered by directed evolution for specific and high affinity to various protein target molecules. These small protein domains are cysteine free, which enables introduction of unique cysteines for thiol mediated site-specific coupling of assorted compounds, and their small size and typically fast and independent reversible folding facilitates engineering of multimeric proteins^11,12^. An Il-17 binding affibody molecule is currently in clinical phase 2 trials (AFFIRM-35) for treating plaque psoriasis (https://clinicaltrials.gov).

In a previous study, we have engineered a high-affinity biparatopic (i.e. a heterodimer of two individual binding entities) affibody molecule that acts as an antagonist to VEGFR2 signaling by blocking VEGFA-binding^13^. Regarding bioactivity, the protein binds to a region on the receptor that is overlapping with the epitope of VEGF-A and was shown to efficiently block VEGF-mediated VEGFR2 phosphorylation, cell proliferation and sprout formation in vitro^14^. The dimer is cross-reactive to both human and murine VEGFR2^14^ and was employed as an in vivo imaging agent for visualization of vascularization in a murine glioblastoma model^15^. Although the previously reported dimer demonstrated promising results both in terms of vascular targeting and agonistic effects, we had identified issues with relatively low thermostability, aggregation and elevated uptake in healthy tissue, most notably liver and spleen, observed in a preclinical biodistribution study^15^.

In addition to high and specific biological activity, the use of proteins in clinical settings requires long shelf-life, high-concentration formulation and retained activity at physiological temperatures. Several of these attributes are directly linked to the thermal stability of the protein but are generally not directly a focus of the initial directed-evolution based drug discovery process of biologics. Furthermore, experience in engineering of proteins has shown that minor alterations in protein sequence can result in dramatic biophysical improvements^16^. However, such properties are generally more difficult to engineer by directed evolution compared with for example affinity and catalytic activity. Numerous more rational protein-engineering strategies have been explored to increase the thermal stability. Examples include approaches based on homology sequence algorithms^17^, high-resolution 3D structures^18^, introduction of disulfide bridges^19^, circularization^20^, shortening of solvent-exposed loops^21^, destabilization of parental protein before engineering^22^, hydrophobic core packing^23^ and mutation of surface-exposed hydrophobic side chains^24^.

Our objective with this study was to improve the thermal stability and refolding capability of the previously developed VEGFR2-binding affibody heterodimer^13^. We explored two different approaches for the engineering: in silico stabilization through a web-based algorithm called Protein Repair One-Stop Shop^25^ (PROSS) and combinatorial directed evolution by an in-house display method based on the gram-positive bacteria *Staphylococcus carnosus*^26^. In staphylococcal display, the recombinant proteins are C-terminally anchored to the peptidoglycan cell wall by endogenous sortase A. The multivalent display (10,000–100,000 copies per cell) enables quantitative assessment of the relative affinity to fluorescently labelled target molecules by high-throughput screening using flow cytometry. In both strategies, the two individual affibody molecules that constitute the dimer were separately engineered as monomers. In parallel, they were subjected to directed evolution and PROSS. The library for the directed evolution approach was designed to exclude new surface-exposed hydrophobic amino acids, which could potentially reduce the hydrophobic surface-area of selected candidates. Output monomers from the two separate tracks were characterized by circular dichroism (CD) spectroscopy and surface plasmon resonance (SPR), to identify stabilizing mutations with retained binding. Stabilized monomers were reformatted into dimers and the biodistribution of the most promising dimeric variant was thereafter evaluated in a murine model.

## Results

### Library design and flow-cytometric screening of libraries

**Figure 1:**
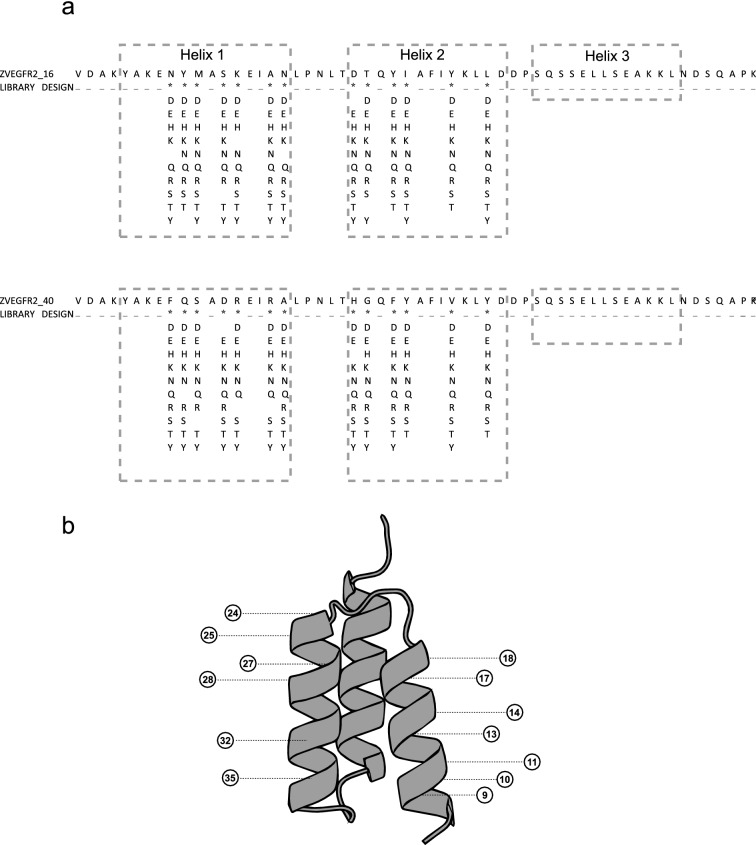
Library design and mutagenesis strategy. (**a**) Original sequences for the two binders and randomized positions with allowed amino acid mutations used to generate the three separate libraries per binder. (**b**) Structure of an affibody molecule (PDB:3MZW) with marked randomized positions in helix 1 and 2.

A total of six libraries were designed for the two original affibody molecules, in which 13 surface-exposed positions were randomized. The mutation frequency was set to 1, 2 and 3 mutations per affibody, respectively, and all hydrophobic residues as well as cysteine and proline were excluded from the randomized positions (Fig. 1). The six libraries are hereinafter denoted 16lib1, 16lib2, 16lib3, 40lib1, 40lib2 and 40lib3. The resulting six individual libraries were separately subcloned into the staphylococcal display vector in fusion to an albumin-binding domain (ABD), which is used for normalization of surface expression level by co-incubation with fluorescently labelled albumin. The subcloned libraries were then transformed into *Staphylococcus carnosus* with diversities covering the library sizes at least ten-fold for each library. DNA sequencing of a subset of clones from respective library confirmed distribution of amino acid mutations and frequencies according to designs (data not shown). Staphylococcal cells displaying affibody molecules with affinity for vascular endothelial growth factor receptor 2 (VEGFR2) were isolated via fluorescence-activated cell sorting (FACS). Sorting gates were set to isolate the top 0.5% of the population with highest target binding to surface expression ratio. Different target concentrations and an off-rate selection was used in the sorting rounds. Only one round of FACS was performed, using a concentration of 100 nM labeled human VEGFR2-His, for the libraries with one mutation per affibody. For the libraries with two and three mutations per affibody, two additional rounds were performed, first with 10 nM labeled human VEGFR2-His and secondly an off-rate selection where the bacterial library was incubated with 100 nM labeled VEGFR2-His, followed by washing and incubation with an excess of unlabeled target for 6 h before sorting (Figure S1). Following the final round of selection, sequencing of variants isolated from the screening revealed unique variants from which the top 5% most frequent sequences for each library were further characterized. The average number of mutations per variant for isolated clones corresponded well to the library design (data not shown).

### Flow cytometric on-cell ranking of VEGFR2 binding

Following FACS, isolated variants with a higher frequency than 5% in the sequenced population were compared by flow-cytometric analysis. Ranking of individual bacteria-displayed binders was done by incubation with labeled VEGFR2-His at 20 nM. A total of 23 clones were analyzed by flow cytometry and ranked by comparing the ratio of VEGFR2 binding signal and surface expression levels. All 23 analyzed clones showed higher normalized signal compared to their non-matured original counterpart (data not shown). The top six candidates from 16lib1-3 and top six from 40lib1-3 with highest signals were chosen for further analysis as soluble proteins (Table 1).

**Table 1:** Selection output variant mutations for Z_VEGFR2_16_ and Z_VEGFR2_40_.

Name	Mutations	# of mutations
Z_VEGFR2_16matC_	D24N, T25Q	2
Z_VEGFR2_16matD_	D24T, T25D	2
Z_VEGFR2_16matE_	D24Q, I28E	2
Z_VEGFR2_16matG_	D24Q, T25S, I28E	3
Z_VEGFR2_16matH_	D24Q, T25H, I28E	3
Z_VEGFR2_16matI_	D24Q, T25Q, I28E	3
Z_VEGFR2_40matB_	Q10Y, S11D	2
Z_VEGFR2_40matC_	S11K, A18S	2
Z_VEGFR2_40matE_	Q10S, S11K	2
Z_VEGFR2_40matH_	Q10R, S11K, F30L	3
Z_VEGFR2_40matI_	Q10K, A18S, H24S	3
Z_VEGFR2_40matL_	Q10S, S11T, A18S	3

### Thermal stability and VEGFR2-binding of affibody monomers isolated from FACS

**Figure 2:**
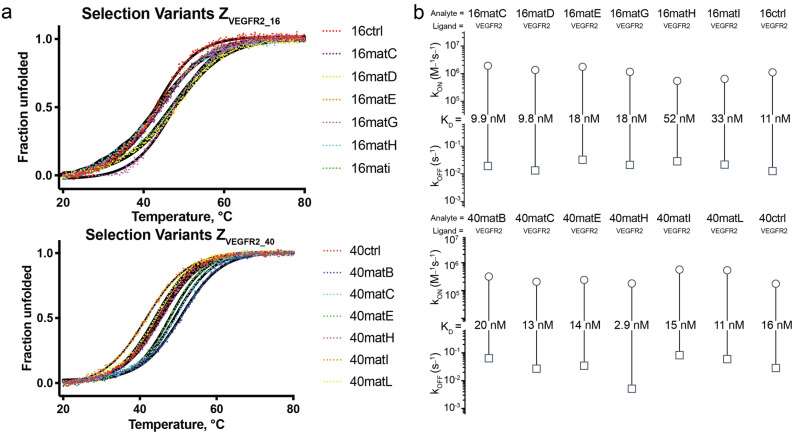
Stability and affinity for monomers isolated from selection. (**a**) Thermal stability of variants from selection, determined by variable temperature CD spectroscopy, shown as fraction unfolded. (**b**) Rate scale plots of variants from selection, affinity constants determined by Biacore 8 K measurement. Rate scale plots were created with www.affinity-avidity.com, provided by Dynamic Biosensors.

Genes encoding the affibody monomers isolated from screening with highest binding signal were subcloned into the vector pET26b( +) for production of soluble proteins in *Escherichia coli*. The proteins were analyzed by circular dichroism (CD) spectroscopy to determine secondary structure content and thermal stability. All clones exhibited alpha-helical content. The melting temperatures of clones isolated from 16lib1-3 varied between 43 and 48 °C and for clones isolated from 40lib1-3 varied between 42 and 49 °C (Fig. 2a). The majority of isolated clones demonstrated both higher melting temperature and more complete refolding than the original binders. Interestingly, a non-intended mutation (F30L) in clone 40matH that is in the hydrophobic core of the protein did not have a negative influence on the thermal stability. The binding of soluble proteins to VEGFR2 was analyzed using surface plasmon resonance (SPR) (Fig. 2b). Data was fitted using non-linear regression to a monovalent binding equation. The binding affinities were determined to be in the range of 10 nM–50 nM for variants isolated from 16lib1-3 and 3 nM–20 nM for variants isolated from 40lib1-3. Interestingly the variant 40matH with the non-intended mutation exhibited the highest affinity. Based on the thermal stability, refolding capability and binding to VEGFR2 two clones were chosen from 16lib1-3 and three from 40lib1-3 for construction of dimers and further analysis.

### In silico design by Protein Repair One-stop Shop (PROSS)

The PROSS tool requires a structure of the protein, which is unavailable for the affibody molecules in this study. Therefore SWISS-MODEL was used to generate a predicted structure based on homology-modeling on a crystal structure of an affibody molecule (3MZW) that binds human epidermal growth factor receptor 2 (HER2). Moreover, PROSS allows locking specific residues in the target protein, i.e. amino acids that are important for binding can be protected from mutations introduced by PROSS. Hence, we locked the 13 residues on the affibody binding interface (in helix 1 and 2) that are usually randomized to generate libraries (Fig. 1). The PROSS generated a total of six variants; three per submitted binder, respectively (Z_VEGFR2_16_ and Z_VEGFR2_40_) (Table 2).

**Table 2:** Pross generated variants for Z_VEGFR2_16_ and Z_VEGFR2_40_ and Z_VEGFR2_16_ with the PROSS6 mutations.

Name	Mutations	# of mutations
Z_VEGFR2_16-PROSS1_	L34N	1
Z_VEGFR2_16- PROSS2_	L34N, S42A, S46A	3
Z_VEGFR2_16- PROSS3_	L34N, S42A, E43N, S46A	4
Z_VEGFR2_40- PROSS4_	E43N	1
Z_VEGFR2_40- PROSS5_	S42A, E43N, S46A	3
Z_VEGFR2_40- PROSS6_	S42A, E43N, S46A, S54A	4
Z_VEGFR2_16- PROSS6_	S42A, E43N, S46A, S54A	4

### Thermal stability and VEGFR2-binding of affibody monomers from PROSS

**Figure 3:**
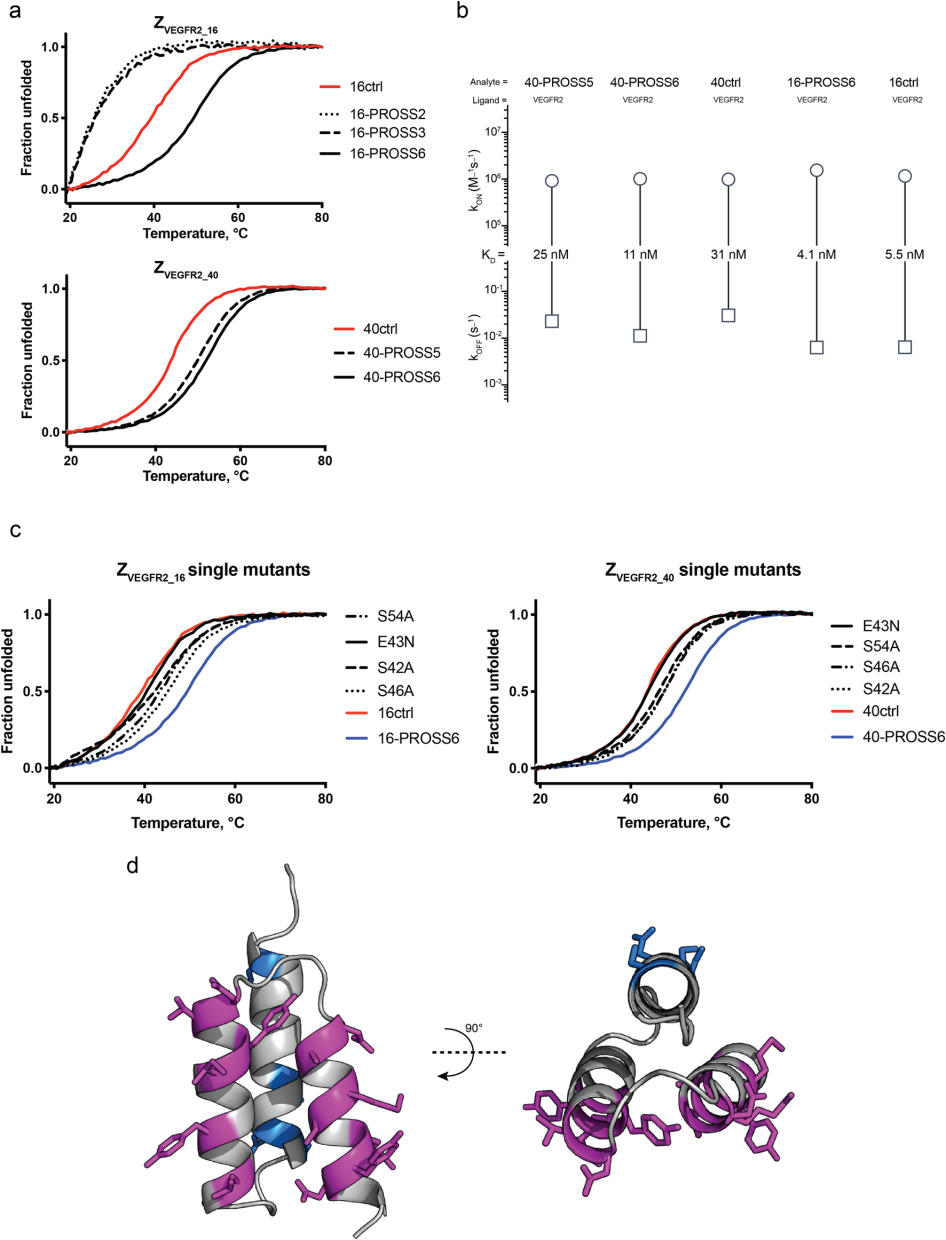
Stability and affinity of monomers generated by PROSS. (**a**) Thermal stability of PROSS variants, determined by variable temperature CD spectroscopy with red representing the original binders. Shown as fraction unfolded. (**b**) Rate scale plots of variants from selection, affinity constants determined by Biacore 8 K measurement. Rate scale plots were created with www.affinity-avidity.com, provided by Dynamic Biosensors. (**c**) Thermal stability of PROSS single-mutant variants, determined by variable temperature CD spectroscopy with red representing the original binders. Shown as fraction unfolded (**d**) Schematic representation of affibody molecule with residue mutations generated by PROSS shown in blue and residues locked to preserve the target-binding interface shown in purple.

Genes for the PROSS-tool generated variants (Z_VEGFR2_16-PROSS2_, Z_VEGFR2_16-PROSS3_, Z_VEGFR2_40-PROSS5_, Z_VEGFR2_40-PROSS6_) (Table 2) were ordered as monomers with a N-terminal His6-tag, cloned into an expression vector and produced in *E. coli*. After purification, we started with analysis of the variants containing multiple mutations (Z_VEGFR2_16-PROSS2_, Z_VEGFR2_16-PROSS3_, Z_VEGFR2_40-PROSS5_ and Z_VEGFR2_40-PROSS6)._ Thermal stability was analyzed by CD spectroscopy and showed that Z_VEGFR2_40-PROSS5_ and Z_VEGFR2_40-PROSS6_ exhibited higher melting temperatures, 6 °C and 9 °C respectively, compared to the original construct (Fig. 3a). On the other hand, Z_VEGFR2_16-PROSS2_ and Z_VEGFR2_16-PROSS3_ had melting temperatures that were too low to be determined (Fig. 3a). This was likely due to the L34N mutation that points into the hydrophobic core of the affibody molecule and probably disrupts the tight packing of the core by introduction of a polar amino acid. The stabilizing mutations from Z_VEGFR2_40-PROSS6_ were therefore grafted onto Z_VEGFR2_16,_ denoted Z_VEGFR2_16-PROSS6_ (Table 2), which resulted in an increase in the melting temperature of 10 °C. Surface plasmon resonance was used to determine the kinetic constants of the mutants and ensure retained ability to bind the receptor (Fig. 3b). In order to determine which mutations from the PROSS6 design that were contributing to increased stability, single mutant variants (i.e. S42A, E43N, S46A and S54A of respective binder) were ordered, produced and characterized by CD as mentioned above. Results showed that all mutations contributed to incremental stabilization in terms of melting temperatures and suggested that the stabilizing mutations are additive (Fig. 3c). A schematic representation of final residue mutations generated by PROSS and amino acids locked from mutation is shown in Fig. 3d.

### Production and size exclusion chromatography (SEC) analysis of new dimeric mutants

The most promising candidates from FACS (Z_VEGFR2_16matD_, Z_VEGFR2_16matG_, Z_VEGFR2_40matB_ and Z_VEGFR2_40matC_) were combined with the most promising mutations from the in silico engineering (i.e. the PROSS6 design). Monomers were then genetically linked with a S4G linker and a C-terminal engineered albumin-binding domain (ABD) was introduced for affinity purification and surface plasmon resonance (SPR) capture assays. We also included Z_VEGFR2_16-40_ (parental dimer) and Z_VEGFR2_16-40-PROSS6_ (parental dimer with PROSS6 mutations in both domains) for comparison. Thus, a total of five constructs were produced (Table 3) as described above and purified by affinity chromatography using the high affinity interaction between ABD and human serum albumin (HSA). Size exclusion chromatography (SEC) was then used to analyze purified dimeric proteins and visualize potential aggregation or degradation (Figure S2). All dimers showed one distinct peak which corresponds to intact dimeric protein. SEC chromatograms for both the original binder Z_VEGFR2_16-40_ and Z_VEGFR2_16-40-PROSS6_ indicated degradation (Figure S2). This tendency of degradation could not be detected in the three new dimeric proteins (Z_VEGFR2_16matD-40matC-PROSS6_, Z_VEGFR2_16matG-40matB-PROSS6_ and Z_VEGFR2_16matG-40matC-PROSS6_).

**Table 3:** Dimeric fusion of monomers from selection output and PROSS6 mutations.

Name	Mutations (Z_VEGFR2_16_//Z_VEGFR2_40_)	# of mutations
Z_VEGFR2_16-40_	–	–
Z_VEGFR2_16-40-PROSS6_	S42A, E43N, S46A, S54A	4
Z_VEGFR2_16matD-40matC-PROSS6_	D24T, T25D, S42A, E43N, S46A, S54A // S11K, A18S, S42A, E43N, S46A, S54A	12
Z_VEGFR2_16matG-40matB-PROSS6_	D24Q, T25S, I28E, S42A, E43N, S46A, S54A // Q10Y, S11D, S42A, E43N, S46A, S54A	13
Z_VEGFR2_16matG-40matC-PROSS6_	D24Q, T25S, I28E, S42A, E43N, S46A, S54A // S11K, A18S, S42A, E43N, S46A, S54A	13

### Affinity determination of new dimeric mutants to VEGFR2

The three different dimers (Z_VEGFR2_16matD-40matC-PROSS6_, Z_VEGFR2_16matG-40matB-PROSS6_ and Z_VEGFR2_16matG-40matC-PROSS6_) were then subjected to biosensor analysis by surface plasmon resonance on a Biacore 8 K to determine their equilibrium dissociation constants (K_D_). Binders were injected over immobilized HSA and captured for subsequent injection of VEGFR2 at varying concentrations. Data was fitted using non-linear regression to a monovalent binding model. Experiments were performed at three temperatures (25, 37 and 40 °C) and obtained affinities were similar between the original dimer and the final stability engineered dimer (Figure S3), demonstrating that the stabilized dimers retained their binding to VEGFR2. All experiments were run in duplicates with freshly prepared reagents.

### Thermal stability of new dimeric mutants

**Figure 4:**
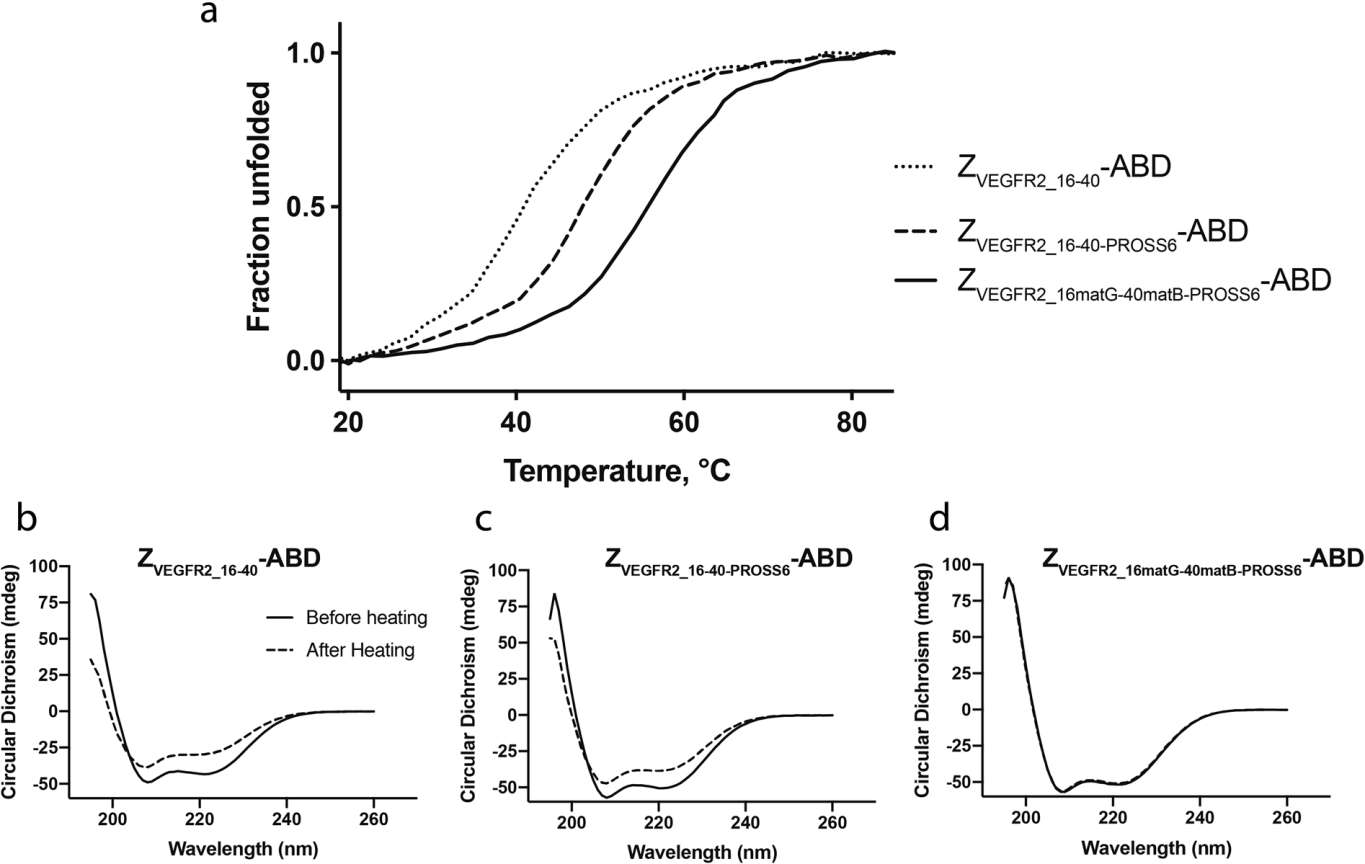
Stability and refolding of dimeric proteins (**a**) Thermal stability of original dimer, PROSS6 mutation dimer and most stable selection/PROSS6 dimer, determined by variable temperature CD spectroscopy. Shown as fraction unfolded. (**b**, **c**, **d**) Refolding capability of original dimer, PROSS6 mutation dimer and most stable selection/PROSS6 dimer.

The three different dimers (Z_VEGFR2_16matD-40matC-PROSS6_, Z_VEGFR2_16matG-40matB-PROSS6_ and Z_VEGFR2_16matG-40matC-PROSS6_) were then further characterized by CD spectroscopy to evaluate their thermostabilities and refolding capabilities. All proteins were subjected to CD analysis at a concentration of 0.3 mg/ml. Thermal stability was determined by measuring ellipticity at 221 nm over a temperature range of 22–90 °C. Lowest stability was seen for the original binder Z_VEGFR2_16-40_. Incorporation of PROSS mutations led to an increase in melting temperature by 7 °C, and the final combination of PROSS mutations with in vitro generated mutations (Z_VEGFR2_16matG-40matB-PROSS6_) led to another 8 °C improvement, adding up to a total of 15 °C improvement in melting temperature (Fig. 4a).

Before and after variable temperature measurement, ellipticity was analyzed at 195–260 nm at 22° for assessment of protein refolding. The original binder Z_VEGFR2_16-40_ and Z_VEGFR2_16-40-PROSS6_ both showed incomplete refolding after variable temperature measurements. Contrarily, the engineered dimer demonstrated complete refolding (Fig. 4b, c, d). The most promising heterodimeric affibody (Z_VEGFR2_16matG-40matB-PROSS6_) is hereinafter denoted Z_VEGFR2_3gen_.

### Production and characterization of protein for radiolabeling

Z_VEGFR2_3gen_ was subcloned with an N-terminal HEHEHE-tag for immobilized metal ion chromatography (IMAC) purification and labelling with [^99m^Tc]Tc(CO)_3_^+^, and produced in *E. coli* as described above. Moreover, a second construct was subcloned and produced in *E. coli* with an N-terminal HEHEHE-tag and also a C-terminal ABD. Produced proteins were characterized by gel electrophoresis, analytical high-performance liquid chromatography (HPLC), electrospray ionization mass spectrometry (ESI–MS) and SPR, which confirmed protein purity, size and VEGFR2-binding, respectively (data not shown).

### Radiolabeling and radiolabel stability

After purification, both proteins were successfully labelled with [^99m^Tc]Tc(CO)_3_^+^ via the HEHEHE-tag. The radiochemical yield was 95% for the dimer and 100% for the ABD-fused dimer as determined by instant thin layer chromatography (ITLC) analysis of reaction mixtures. Isolated yield was 76 ± 1% and 89.5 ± 0.5%, respectively. Radiochemical purity of labeled conjugates used in the experiments was 100%. Label stability was analysed after 4 h incubation in PBS and both conjugates demonstrated high stability (over 99% of activity was associated with protein).

### Analysis of in vitro specificity and cellular processing

**Figure 5:**
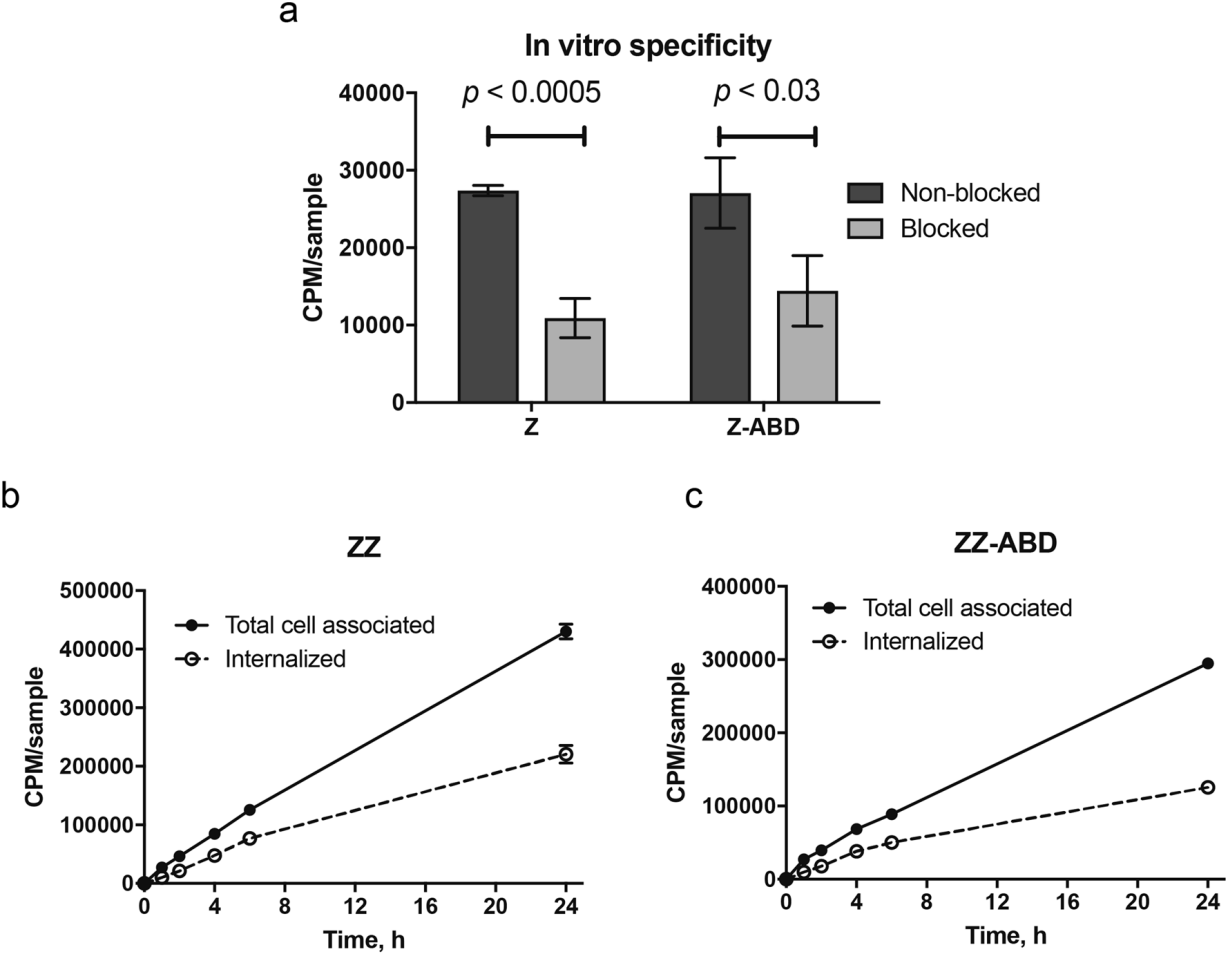
In vitro binding specificity and internalization rate. (**a**) In vitro binding specificity of [^99m^Tc]Tc(CO)3-HEHEHE-Z_VEGFR2_3gen_ (ZZ) and [^99m^Tc]Tc(CO)3-HEHEHE-Z_VEGFR2_3gen_-ABD (ZZ-ABD) tested on MS1 cells in the presence or absence of non-labelled conjugate. The cell-associated activity is presented as CPM per dish (average value from three cell dishes ± SD). (**b**, **c**) Binding and internalization of [^99m^Tc]Tc(CO)_3_-HEHEHE-Z_VEGFR2_3gen_ (ZZ) and [^99m^Tc]Tc(CO)_3_-HEHEHE-Z_VEGFR2_3gen_-ABD (ZZ-ABD) by MS1 cells. Data are presented as average values from three cell dishes ± SD. Error bars might not be visible because they are smaller than point symbols.

Analysis of binding of both labeled conjugates to murine VEGFR2-expressing cells (MS-1) showed that cell-associated activity was significantly lower for cells pre-incubated with non-labelled conjugate, indicating specific binding (Fig. 5a). Cellular processing of labelled conjugates over time was studied using MS-1 cells under continuous incubation. Both conjugates demonstrated similar patterns of binding and internalization (Fig. 5b,c). Cell-associated activity increased over time, the internalization of labelled conjugates was moderate and internalised fraction reached 50% for [^99m^Tc]Tc(CO)_3_-HEHEHE- Z_VEGFR2_3gen_ and 40% for [^99m^Tc]Tc(CO)_3_-HEHEHE- Z_VEGFR2__3gen-ABD at the end of the observation period.

### In vivo biodistribution studies

**Figure 6:**
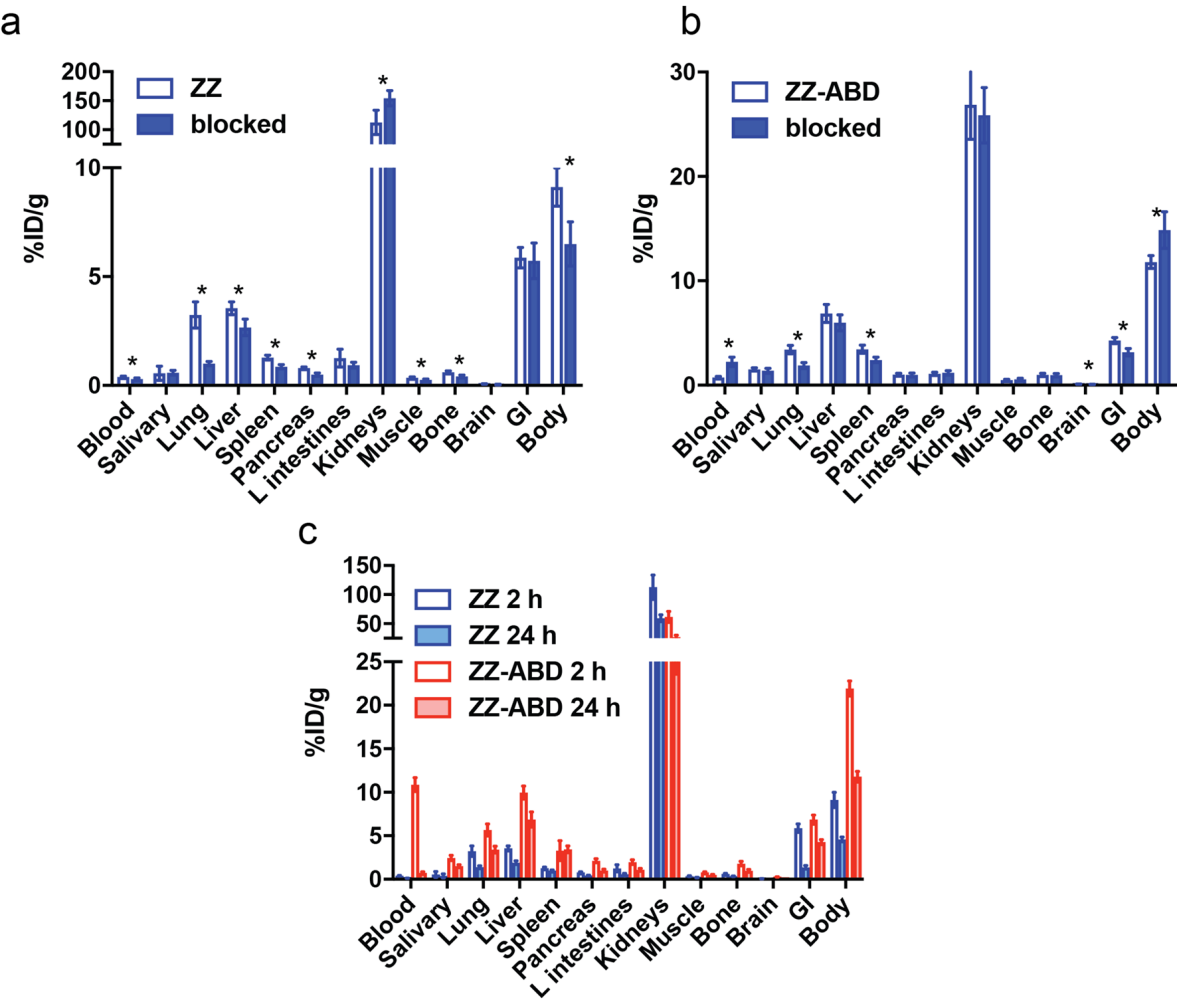
Biodistribution data of Z_VEGFR2_3gen_. (**a**, **b**) Biodistribution of [^99m^Tc]Tc(CO)_3_-HEHEHE-Z_VEGFR2_3gen_ (ZZ) 2 h pi and [^99m^Tc]Tc(CO)_3_-HEHEHE-Z_VEGFR2_3gen_-ABD (ZZ-ABD) 24 h pi in NMRI mice. Data are presented as average values ± SD (n = 4). Injected protein doses were 0.3 nmol and 3 nmol for [^99m^Tc]Tc(CO)_3_-HEHEHE-Z_VEGFR2_3gen_ and 0.3 nmol and 6 nmol for [^99m^Tc]Tc(CO)_3_-HEHEHE-Z_VEGFR2_3gen_-ABD (higher dose is designated as blocked). c) Biodistribution of [^99m^Tc]Tc(CO)_3_-HEHEHE-Z_VEGFR2_3gen_ (ZZ) and [^99m^Tc]Tc(CO)_3_-HEHEHE-Z_VEGFR2_3gen_-ABD (ZZ-ABD) in NMRI mice 2 and 24 h pi. Data are presented as average values ± SD (n = 4). Injected protein dose was 0.3 nmol.

Biodistribution of [^99m^Tc]Tc(CO)_3_-HEHEHE-Z_VEGFR2__3gen and [^99m^Tc]Tc(CO)_3_-HEHEHE- Z_VEGFR2__3gen-ABD was investigated in NMRI mice using two different protein doses, respectively (Fig. 6a,b). Both conjugates demonstrated dose-dependent distribution patterns. For organs with endogenous receptor expression (e.g. lung, liver and spleen), injection of higher protein dose (i.e. more unlabelled affibody constructs) lead to a significant decrease in activity uptake (for ABD-fused conjugate not significant, but strong tendency for liver), indicating blockable receptor-mediated uptake. Activity uptake in salivary gland (i.e. organs that typically accumulate radiocatabolites in case of label instability) was low for both injected protein doses, which indicate high in vivo stability of radiolabels.

Activity accumulation in blood, kidney and whole body were influenced differently for the neat dimer and the ABD-fused dimer when protein dose was increased. For the dimer, activity uptake in blood and whole body decreased and activity uptake in kidneys increased. For the ABD-fused dimer, activity uptake in blood and whole body significantly increased.

Biodistribution patterns of [^99m^Tc]Tc(CO)_3_-HEHEHE-Z_VEGFR2_3gen_ and [^99m^Tc]Tc(CO)_3_-HEHEHE- Z_VEGFR2_3gen_-ABD were compared 2 and 24 h pi in normal mice (Fig. 6). Dimer without ABD demonstrated rapid blood clearance that was similar to the previously studied biparatopic dimer^15^. Comparison of the biodistribution patterns of the new conjugates 2 h pi clearly demonstrated that ABD-fused dimer had significantly longer blood circulation and lower excretion rate than non-fused dimer.

The ABD-fused conjugate demonstrated 20-fold higher activity concentration in blood in comparison with non-fused dimer at 2 h pi. In agreement with this result, activity uptake in all tested organs and tissues was higher for [^99m^Tc]Tc(CO)_3_-HEHEHE-Z_VEGFR2_3gen_-ABD, except for kidneys where activity uptake was significantly lower for ABD-fused dimer. The overall pattern of activity uptake was similar at both time points for both tested conjugates. With time, initial activity uptake decreased in all studied tissues (values were significantly lower for all tested organs and tissues except salivary glands for non-fused conjugate and spleen for ABD-fused one).

Comparison of blood retention of ABD-fused dimer with other ABD-fused affibody molecules showed that blood clearance of VEGFR2-targeting affibody conjugate was more rapid than HER2-targeting [^111^In]In-Z_HER2:2891_-ABD_035_-DOTA^27^, but comparable with some HER3-targeting conjugates, particularly [^111^In]In-(Z_HER3:08698_)_2_-ABD_035_-DOTA and [^111^In]In-ABD_035_-(Z_HER3:08698_)_2_-DOTA^28^.

## Discussion

Here, we report on the engineering and evaluation of a recently generated so-called “biparatopic” affibody molecule that binds two adjacent and non-overlapping sites within the VEGF-binding epitope on human and murine VEGFR2 with picomolar affinity. The dimeric affibody binds to the VEGF epitope on the receptor and has been shown to be an efficient agonist by inhibiting ligand-induced phosphorylation and sprouting of endothelial cells^14^. In a previous study, [^111^In]In-labeled affibody dimer was able to specifically target VEGFR2 in vivo with much higher tumor-to-blood ratios compared to other VEGFR2 imaging probes^15^. However, the [^111^In]In-labeled constructs use is challenging due to relatively high uptake in some metastatic sites such as liver and spleen. Furthermore, the affibody construct demonstrated issues related to relatively low thermostability, aggregation tendencies, incomplete refolding and high uptake in liver and spleen^15^. The latter has been shown to correlate with the overall hydrophobicity of affibody-based tracers^29^. Thus, the overall aim of this study was therefore to engineer new dimeric variants with increased thermal stability and solubility, while retaining high-affinity binding to VEGFR2.

We here used two protein engineering strategies_._ In each track, affibody monomers constituting the dimer were engineered separately. A web-based in silico modelling algorithm called PROSS was used to target framework residues (i.e. amino acids not involved in receptor binding), and directed evolution by an in-house staphylococcal display method and FACS was used to target residues in the binding interface. The libraries for directed evolution were designed with the aim to increase hydrophilicity while retaining binding.

Mutations proposed by the PROSS algorithm resulted in stabilizing mutations for Z_VEGFR2_40_. On the other hand, initial PROSS mutations suggested for Z_VEGFR2_16_ introduced a polar side chain (L34N) into the hydrophobic core of the protein, which turned out to be detrimental for the stability of the protein. Mutations for Z_VEGFR2_40_, which demonstrated the highest thermal stabilization, were therefore grafted onto Z_VEGFR2_16_, which resulted in similar stabilization. Interestingly, single-mutant analysis demonstrated that suggested mutations were predominantly additive, a feature mentioned in the development of PROSS^25^.

A number of new variants were isolated by FACS from the six libraries, and CD spectroscopy and SPR revealed that the vast majority had improved thermal stability and retained high-affinity binding to VEGFR2. The mutations from PROSS and FACS were thus combined into new versions of the monomers, which were then fused again to construct new dimeric binders. The final dimeric protein (Z_VEGFR2_3gen_) had a 15 °C improvement in melting temperature and demonstrated complete refolding after heat treatment, in contrast to the original binder.

The biodistribution of Z_VEGFR2_3gen_ was finally evaluated in NMRI mice. The most striking difference when comparing with previous biodistribution studies of the original binder, was the rather dramatic decrease in activity uptake in spleen and liver^15^, which is likely due to the increased thermostability and complete refolding for the new engineered binder. The new conjugate [99mTc]Tc(CO)3-HEHEHE-Z_VEGFR2_3gen_ showed approximately 13-fold lower uptake in liver and spleen compared to the previous version of the dimeric VEGFR2-specific affibody^15^. It has been shown in previous biodistribution studies with affibodies that the hydrophobicity of the tracer has a significant impact on uptake in liver and spleen^29^. However, it should be noted that different radio labelling strategies were used in these two studies, which might influence the biodistribution. From the biodistribution results, it was also evident that fusion of the engineered ABD lead to a dramatic increase in serum half-life of the affibody construct, indicating that this would be a feasible strategy to prolong the blood circulation time in future therapeutic studies. Comparison of blood retention of ABD-fused dimer with other ABD-fused affibody molecules showed that blood clearance of VEGFR2-targeting affibody conjugate was more rapid than HER2-targeting [^111^In]In-Z_HER2:2891_-ABD_035_-DOTA^27^, but comparable with some HER3-targeting conjugates, particularly [^111^In]In-(Z_HER3:08698_)_2_-ABD_035_-DOTA and [^111^In]In-ABD_035_-(Z_HER3:08698_)_2_-DOTA^28^.

To conclude, we have used directed evolution and an algorithm-based approach to generate VEGFR2 binding affibody molecules with increased stability. The stabilizing mutations that were identified from the two different procedures were ultimately grafted into one construct and fused in a dimeric format to generate variants with significantly enhanced stability. Retained binding of the affibody molecules to VEGFR2 was verified by SPR. A biodistribution study also indicated decreased uptake in healthy tissues and these results together with previously published data on strong agonistic activity show that the VEGFR2-specific affibody molecules are promising for further in vivo studies and evaluations.

## Materials and methods

### Design and molecular cloning of combinatorial libraries

A total of six different libraries were designed and ordered from Twist bioscience (Fig. 1a). The genes were amplified by eight cycles of PCR using Phusion DNA polymerase, purified by QIAquick PCR purification kit (Qiagen, GmbH, Hilden, Germany) and digested with restriction enzymes XhoI and SacI. Digested oligonucleotides and plasmid (pSCZ1) were purified by preparative gel electrophoresis (2% GTG agarose) and Qiaquick gel extraction kit (Qiagen GmbH). Libraries were ligated into the display vector using T4 DNA ligase (New England Biolabs, Beverly, MA, USA), according manufacturer’s instructions. Ligated plasmids were purified and concentrated using ethanol precipitation before being transformed into electrocompetent One Shot TOP10 cells, which were used as a host for plasmid production.

Preparation was performed with a JETSTAR Maxi Kit (Genomed, Bad Oeynhausen, Germany) according to manufacturer’s instructions and concentrated by ethanol precipitation. Plasmids were sequenced (Microsynth AG, Balgach, Switzerland) before transformation into *Staphylococcus carnosus* TM300 by electroporation as previously described^30^. Individual colonies were then once again sequenced (Microsynth, Balgach, Switzerland) prior to selection screening.

### FACS of bacteria-displayed affibody libraries

All six libraries were individually inoculated to 5 ml tryptic soy broth supplemented with yeast extract (TSB + YE; Merck) with 10 μg/ml chloramphenicol (Sigma-Aldrich) and incubated at 150 rpm 37 °C ON. The next day, cells were harvested by centrifugation (3000 g, 5 min, RT) and labelled for flow cytometry as previously described^31^ using biotin-XX Microscale Protein Labeling Kit biotinylated (ThermoFisher Scientific, Massachusetts, USA) VEGFR2-His (Sino Biological Inc., Beijing, China) in a first incubation of 2 h, and secondary Streptavidin R-phycoerythrin conjugate (SAPE; Invitrogen) and human serum albumin conjugated to Alexa Fluor 647 (HSA-Alexa Fluor 647) for 45 min in a second incubation. Before screening, cells were washed three times in ice-cold phosphate-buffered saline supplemented with 0.1% Pluronic F108 NF Surfactant (PBSP; pH 7.4; BASF Corporation, Mount Olive, NJ) and resuspended in 1.5 ml ice-cold PBSP. One round of selection, with target VEGFR2 concentration of 100 nM, was performed for the smallest libraries containing only one mutant per variant and three rounds were performed for the libraries containing 2 and 3 mutations per variant (Figure S1), using a MoFlo Astrios flow cytometer (Beckman Coulter, Indianapolis, IN, USA). Around 0.5% of the population was sorted in each round based on highest surface expression-normalized VEGFR2-binding signal, into TSB + YE and grown overnight with 10 μg/ml chloramphenicol. In the second round of selection, a target concentration of 10 nM was used. In the third round, an off-rate selection strategy was applied where cells were first incubated with 100 nM VEGFR2-His and after washing, an excess of unlabeled VEGFR2 was added and left for 6 h incubation, before secondary labeling. Finally, cells isolated from selections were plated on chloramphenicol containing plates and individual colonies were sequenced (Microsynth).

### Analysis of VEGFR2 binding of isolated clones by flow cytometry

Individual clones isolated from the selection were inoculated in 5 ml TSB + YE with 10 μg/ml chloramphenicol and grown ON at 150 rpm and 37 °C. Cells were pelleted by centrifugation at 3000 g and re-suspended in PBSP containing biotinylated VEGFR2. After 1 h, cells were washed with ice-cold PBSP and re-suspended in ice-cold PBSP containing SAPE and HSA-Alexa Fluor 647. Finally, cells were washed twice in ice-cold PBSP and analyzed by flow-cytometry using a Gallios flow cytometer (Beckman Coulter). Cells were then ranked based on ratio between VEGFR2-binding and cell surface expression (Figure S1). All experiments were performed in duplicates on different days using freshly prepared reagents.

### Subcloning and production of affibody molecule variants from selection

A total of twelve monomeric variants isolated from the screening were subcloned from the pSCZ1 display vector into pET-26b( +). Soluble protein production in *Escherichia coli* BL21 Star (DE3) cells (Invitrogen) and purification by immobilized metal ion chromatography (IMAC) was performed as previously described^13^.

### Circular dichroism spectroscopy

All circular dichroism spectroscopy experiments were performed by subjecting affibody molecules to 195–260 nm and 22 °C using a Chirascan spectrometer (Applied Biosystems, California, USA) in a cell with an optical path-length of 1 mm at a concentration of 0.3 mg/ml. Thermal stability was analyzed by measuring ellipticity at 221 nm over a temperature range of 22–90 °C (5 °C/min).

### Surface plasmon resonance characterization of monomers isolated from selection

Surface plasmon resonance (SPR) experiments were performed using a Biacore T200 instrument (GE Healthcare, Uppsala, Sweden) using phosphate-buffered saline supplemented with 0.05% Tween 20 (PBST 0.05) as running buffer and 10 mM HCl for regeneration. Signal from blank surface interactions were subtracted for each run. Recombinant human VEGFR2-His (Sino Biological) was immobilized by amine coupling on two surfaces of a CM5 sensor chip (GE Healthcare). Binding of variants isolated from library screening was analyzed by injections of five different concentrations of the Affibody molecules (100, 50, 25, 12.5 and 6.25 nM) over the immobilized VEGFR2-His. The experiment was performed in duplicates using freshly prepared reagents.

### Protein Repair One-stop Shop (PROSS)

Following the instructions on the web server (https://pross.weizmann.ac.il), two homology modeled protein structures based on Z_VEGFR2_16_ and Z_VEGFR2_40_ were generated from PDB:id 3MZW and submitted to the PROSS. 13 amino acids that are part of the binding interface on the affibody molecule in helix 1 and 2 were locked whilst remaining residues were subject to introduction of mutations by PROSS. The sequence alignment required for the PROSS to function was generated through the website’s default process.

### Molecular cloning, protein production and purification of PROSS-generated variants

Genes for the variants generated by PROSS were synthesized and sub-cloned into the expression vector pET26b( +) by using XhoI and NdeI restriction sites (GeneArt, ThermoFischer Scientific, Courtaboeuf, France). A His6-tag was included N-terminally to facilitate purification by immobilized metal ion chromatography (IMAC). Purified proteins were characterized as described above, by CD to determine thermostability and SPR to determine affinity towards VEGFR2.

### Subcloning, expression and purification of dimeric binders

Monomeric variants isolated from the library with the highest stability and retained binding were grafted together with the stabilizing mutations generated by PROSS, and an engineered albumin-binding domain (ABD) was added to facilitate purification and surface plasmon resonance capture-based analysis. The genes for five different dimeric constructs (Table 3) were ordered from ThermoFischer Scientific and subcloned into the expression vector pET26b ( +), transformed to *E. coli* BL21 Star (DE3) cells (Invitrogen), and produced and purified as described above.

### Radiolabelling and stability

All buffers used for labelling and purification were prepared from chemicals from Sigma-Aldrich. Site-specific radiolabelling of HEHEHE-ZVEGFR2_3gen and HEHEHE-ZVEGFR2_3gen-ABD with [^99m^Tc]Tc(CO)_3_(H2O)_3_]^+^ was performed as described earlier^32^. Briefly, the generator eluate (500 μl) containing ∼4 GBq of [^99m^Tc]TcO_4_^−^ was added in a sealed vial containing a CRS kit (Center for Radiopharmaceutical Sciences, PSI, Villigen, Switzerland) and incubated at 100 °C for 30 min. After incubation, 40 μl of technetium tricarbonyl solution was added to conjugate solutions in PBS (6.8 nmol, 1 mg/ml)). The reaction was incubated for 60 min at 50 °C. The labelling yield was determined by instant thin layer chromatography (ITLC; 150–771 DARK GREEN, Tec-Control Chromatography strips, Biodex Medical Systems) eluted with PBS. Radio-ITLC were measured on a Cyclone Storage Phosphor System on radioactivity distribution and analysed with the OptiQuant image analysis software (both from Perkin Elmer Sweden AB). The radiolabelled conjugates were purified using NAP-5 columns (Amersham Biosciences) eluted with PBS. The in vitro stability test was performed by incubating the radiolabelled conjugates in PBS for 4 h at room temperature. The stability was assessed as described above.

### In vitro characterization

The pancreatic islet endothelial cells MS1 (Mus EC MS1 mouse endothelial) used was a kind gift from Dr. Jack L. Arbiser, Children’s Hospital, Harvard Medical School, Boston, MA^33^.

MS1 cells were cultured in DMEM growth media (Dulbecco’s Modified Eagle Medium) with a low L-glutamine level, supplemented with 10% FBS (Fetal bovine serum, Sigma-Aldrich) and 1% PEST (Penicillin 100 UI/mL, streptomycin 100 µg/mL, all from Biochrom AG) in a 37 °C incubator with 5% CO_2_ as described earlier^15^. Harvesting of cells was performed by treatment with 0.25% trypsin, 0.02% EDTA solution (Biochrom AG). Change of media was done twice a week, and the cells were seeded one day prior to experiments. Experiments were done in triplicate.

In vitro characterization of [^99m^Tc]Tc(CO)_3_-HEHEHE- Z_VEGFR2__3gen and [^99m^Tc]Tc(CO)_3_-HEHEHE- Z_VEGFR2__3gen-ABD was performed as described earlier^15^. Briefly, for in vitro binding specificity test MS-1 cells were incubated with 1 nM solution of labelled conjugates with or without pre-incubation with 100 nM solution of non-labelled conjugates for 1 h 37 °C. Cells were harvested and measured on activity content using an automated gamma well counter (3-inch NaI(Tl) detector, 2480 Wizard^2^, PerkinElmer).

Further, to study cellular processing, MS-1 cells were incubated with 1 nM solution of labelled conjugates as described earlier^15^. At predetermined time points (up to 24 h), a set of dishes was treated with 0.5 mL of acid buffer (4 M urea in 0.2 M glycine buffer, pH 2.0) on ice for 5 min. The acid fraction was considered to represent the membrane bound conjugate. A base solution (NaOH, 1 M, 0.5 mL) was added to the cells and incubated for 30 min at 37 °C. The base solution was considered as the internalized conjugate. Activity in samples was measured as described above.

### In vivo experiments

All animal experiments were planned and performed in accordance with national legislation on laboratory animals’ protection and were approved by the Ethics Committee for Animal Research in Uppsala. The mice were sacrificed by intraperitoneal injection of a Ketalar-Rompun solution (10 mg/mL Ketalar and 1 mg/mL Rompun; 20 μl of solution per gram of body weight). The tissue samples were collected for radioactivity measurements. The gastrointestinal tract with content and the rest of carcass were also collected. Activity of samples was measured together with injection standard as described above.

Female NMRI mice (n = 4) were used to study biodistribution of [^99m^Tc]Tc(CO)_3_-HEHEHE- Z_VEGFR2__3gen and [^99m^Tc]Tc(CO)_3_-HEHEHE- Z_VEGFR2__3gen-ABD at 2 and 24 h pi. Labelled conjugates were iv injected in equimolar amounts in PBS (0.3 nmol/animal in 100 µl, 30 kBq/animal at euthanisation). Additionally, one group for each conjugate was injected with higher protein dose, 3 nmol/animal for [^99m^Tc]Tc(CO)_3_-HEHEHE- Z_VEGFR2__3gen and 6 nmol for [^99m^Tc]Tc(CO)_3_-HEHEHE- Z_VEGFR2__3gen-ABD. Biodistribution in these groups was studied at 2 h pi for [^99m^Tc]Tc(CO)_3_-HEHEHE- Z_VEGFR2__3gen and 24 h pi for [^99m^Tc]Tc(CO)_3_-HEHEHE- Z_VEGFR2__3gen-ABD. Sedated animals were sacrificed by heart puncture after i.p. injection of anaesthesia solution. Dissected organs and tissue samples were measured on activity content as described above.

### Statistical analysis

Statistical analysis was performed using Prism 8.1.1 software (GraphPad Software Inc). A 2-tailed unpaired t-test was applied to find a significant difference for comparison of two sets of data.

## Supplementary information

Supplementary Information.

## Data Availability

The data generated during and/or analysed during the current study are available from the corresponding author on reasonable request.
