# Supplementary material for: Increasing thermal stability and improving biodistribution of VEGFR2-binding affibody molecules by a combination of in silico and directed evolution approaches
Source: Sci Rep. 2020 Oct 23;10:18148. doi: 10.1038/s41598-020-74560-5 (PMC7585445; doi:10.1038/s41598-020-74560-5)
Supplement: Supplementary file 1 — Supplementary Information. [file 41598_2020_74560_MOESM1_ESM.pdf]

Supporting information

Increasing thermal stability and improving biodistribution of VEGFR2-binding affibody molecules by a combination of in silico and directed evolution approaches

Rezan Güler[1], *Siri Flemming Svedmark*[1], *Ayman Abouzayed*[2], *Anna Orlova*[2,3,4], *John Löfblom*[1]*

[1]Department of Protein Science, School of Engineering Sciences in Chemistry, Biotechnology and Health, KTH Royal Institute of Technology, Stockholm, Sweden.

[2]Department of Medicinal Chemistry, Uppsala University, Uppsala, Sweden

[3]Science for Life Laboratory, Uppsala University, Uppsala, Sweden

[4]Research Centrum for Oncotheranostics, Research School of Chemistry and Applied Biomedical Sciences, Tomsk Polytechnic University, Tomsk, Russia

*Corresponding author: John Löfblom; Telephone: +46 8 790 9659; E-mail: lofblom@kth.se.

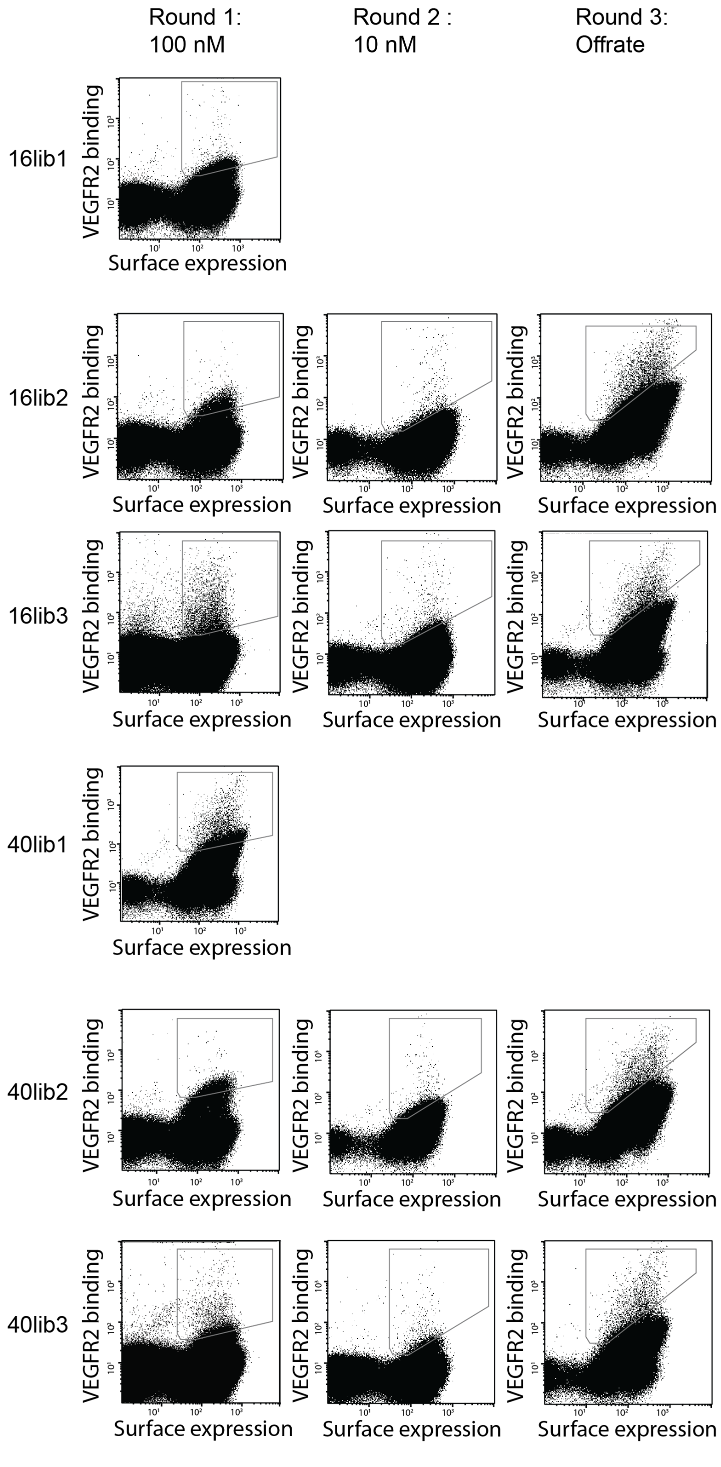

Figure S1. Library sort rounds and FACS plots with Y-axis signal corresponding to VEGFR2 binding and X-axis signal to surface expression. 16lib2/3 and 40lib2/3 were screened against decreasing target concentration followed by an off-rate selection in presence of excess unlabeled target. Approximate gating for selection rounds are indicated.

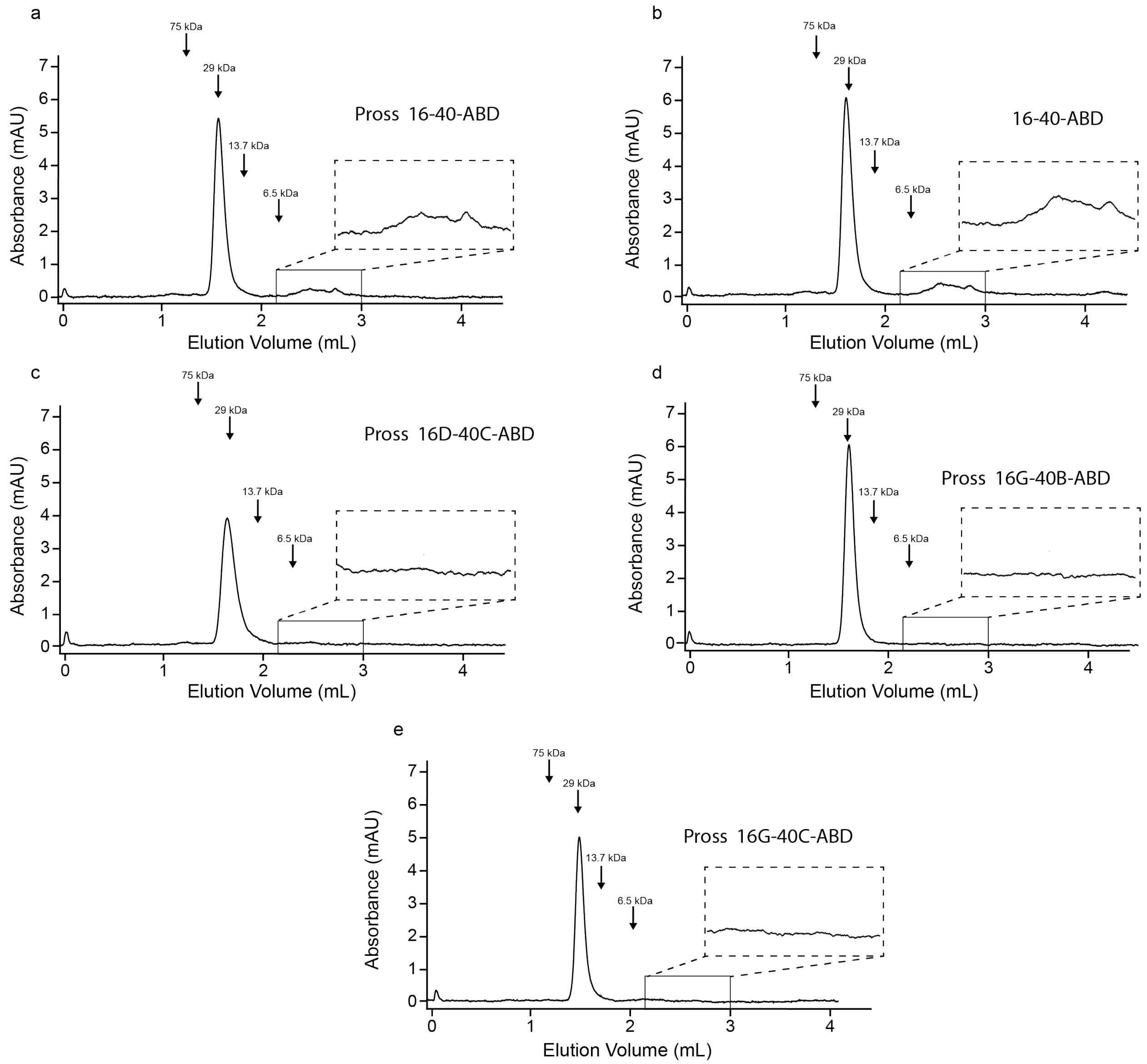

Figure S2. Size exclusion chromatography of dimeric proteins. Arrows indicate elution points of standard proteins.

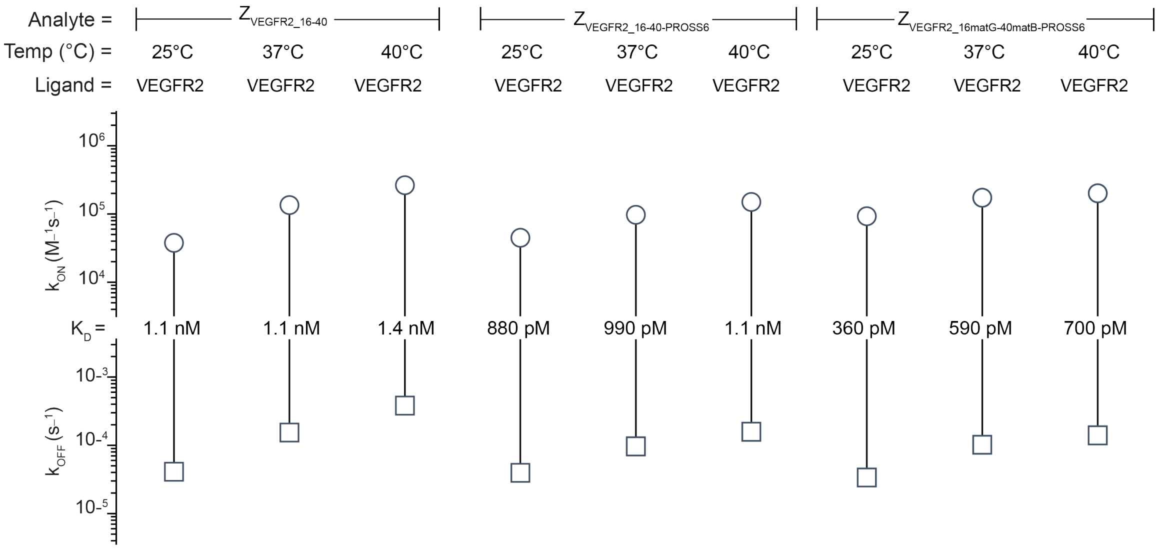

Figure S3. Rate scale plots of original dimer, PROSS-mutated dimer and the most stable selection/PROSS dimer variants at three different temperatures: 25, 37 and 40°C. Affinity constants were determined by Biacore 8K measurements. Values shown are averages of duplicate runs. Rate scale plots were created with www.affinity-avidity.com, provided by Dynamic Biosensors.
